# Fungal endophytes in germinated seeds of the common bean, *Phaseolus vulgaris*

**DOI:** 10.1016/j.funbio.2016.01.017

**Published:** 2016-05

**Authors:** Soroush Parsa, Adriana M. García-Lemos, Katherine Castillo, Viviana Ortiz, Luis Augusto Becerra López-Lavalle, Jerome Braun, Fernando E. Vega

**Affiliations:** a Life Sciences Innovation Center, University of California, Davis – Chile, Andrés Bello 2299 No. 1102, Providencia, Santiago, Chile; b Centro Internacional de Agricultura Tropical (CIAT), Apartado Aéreo 6713, Cali, Colombia; c Statistical Consultant, 3034 Boulder Place, Davis, CA 95618, USA; d Sustainable Perennial Crops Laboratory, United States Department of Agriculture, Agricultural Research Service, Building 001, BARC-W, Beltsville, MD 20705, USA

**Keywords:** *Aureobasidium pullulans*, Biological control, Endophytic, Fungal biology, Seed-borne fungi

## Abstract

We conducted a survey of fungal endophytes in 582 germinated seeds belonging to 11 Colombian cultivars of the common bean (*Phaseolus vulgaris*). The survey yielded 394 endophytic isolates belonging to 42 taxa, as identified by sequence analysis of the ribosomal DNA internal transcribed spacer (ITS) region. *Aureobasidium pullulans* was the dominant endophyte, isolated from 46.7 % of the samples. Also common were *Fusarium oxysporum*, *Xylaria* sp., and *Cladosporium cladosporioides*, but found in only 13.4 %, 11.7 %, and 7.6 % of seedlings, respectively. Endophytic colonization differed significantly among common bean cultivars and seedling parts, with the highest colonization occurring in the first true leaves of the seedlings.

## Introduction

Plant seeds internally host a diversity of microorganisms that may be transmitted locally or systemically to the developing plant (Mano et al., 2006, Rijavec et al., 2007, Ferreira et al., 2008, Kaga et al., 2009). When the microorganism does not cause any apparent symptoms in the plant it is called an endophyte (Hyde & Soytong 2008). Endophytes are ubiquitous in nature, and some have shown potential to enhance their host's growth, tolerance to abiotic stress, or resistance to pests and pathogens (Wani *et al.* 2015). For this reason, significant and growing interest surrounds their application in agriculture (Hallmann et al., 1997, Backman and Sikora, 2008). Exploring this potential, our study sought to identify promising fungal endophytes naturally occurring in germinated seeds of the common bean, *Phaseolus vulgaris*.

The common bean is the most important legume crop consumed by humans worldwide (Broughton *et al.* 2003). It is grown in over 12 million hectares and feeds more than 500 million people in Latin America and Africa alone (Schwartz & Corrales 1989). This crop is also significantly constrained by biotic and abiotic stressors, top among them plant pathogens and drought (Schwartz and Corrales, 1989, Allen et al., 1996). Partly due to these constraints, bean yields in developing countries average ca. 650 kg ha^−1^, roughly 35 % of the yield achieved in the US and Canada (Singh 1999). Exploring the utility of endophytes as biocontrol agents to increase bean production is therefore well justified.

Laboratory studies are beginning to unveil the potential of fungal endophytes for common bean production. In one of these studies, endophytic *Trichoderma* has been found to stimulate common bean growth (Hoyos-Carvajal *et al.* 2009). More intriguingly, when established as a root endophyte, the fungal entomopathogen *Metarhizium robertsii* was shown to translocate nitrogen from a dead insect to a common bean plant host, suggesting this endophyte's potential to protect its host plant from soil pests and at the same time promote plant growth (Behie et al., 2012, Behie and Bidochka, 2014). Also promisingly, root colonization by *Glomus intraradices*, an arbuscular mycorrhizal fungus, has been shown to protect the common bean from dehydration caused by drought and high salinity (Aroca *et al.* 2007) while another arbuscular mycorrhizal fungus, *Glomus macrocarpum*, stimulates common bean nodulation and growth (Daft & El-Giahmi 1974).

Little is known about other fungal endophytes naturally occurring in common bean seeds. A recent search for seed-borne bacterial endophytes in the common bean yielded over 50 species, including the new species *Rhizobium endophyticum* (López-López *et al.* 2010). A similar search for fungal endophytes is therefore warranted. We responded to this imperative by screening common bean seeds from 11 cultivars grown in Colombia, an important center of diversity for this crop. Our objective was to identify seed-borne fungal endophytes that are transmitted to seedlings and have the potential to enhance common bean production.

## Materials and methods

### Seed samples and germination

We obtained 1120 seeds representing 11 common bean cultivars from the Genetic Resources Unit at the International Center for Tropical Agriculture (CIAT, after its Spanish acronym) and from a local supermarket in Palmira, Colombia (Table 1). Sixty to 100 seeds of each cultivar were surface sterilized by immersion in 0.1 % Triton X-100 (SIGMA, St. Louis, MO) for 2 min, followed by 0.5 % sodium hypochlorite for 2 min, and 70 % ethanol for 2 min. The seeds were then rinsed three times in sterile distilled water and dried in sterile towel paper. The effectiveness of the seed surface sterilization method was evaluated by pressing individual seeds unto 100 mm × 15 mm Petri dishes containing potato dextrose agar (PDA; Difco™, Sparks, MD) and incubating the plates at 26 °C for 10 d. The disinfection was considered successful when no fungal growth was observed in the PDA plate by the end of the incubation period. The sample was discarded if fungal growth was positive.

Each surface sterilized seed was individually planted in a 50 cm^3^ sterilized germination tray cell (PlastiKa Asociados Ltda., Bogotá, Colombia), containing 11 g of autoclaved vermiculite moistened with 18 ml of sterile distilled water. The plants were allowed to grow for eight days in a walk-in growth chamber set at 25 °C, 47 % relative humidity (RH) and a 12 h photoperiod (10 000 lux). All surfaces of the growth chamber were disinfected with the antimicrobial product MonoFoil M1 (Coeus Technology, Anderson, IN) and 70 % ethanol before placing the germination trays inside the chamber. Plants were watered with 8 ml of sterile distilled water on days 3, 5, 6, and 7 after planting. To monitor airborne fungal spores that could infect seedlings in the growth chamber, Petri dishes containing PDA media were periodically exposed as sentinels for 15 min inside the growth chamber, incubated for 10 d at 26 °C, and any ensuing fungal growth characterized morphologically. Although a valuable monitoring tool, this method cannot guarantee the complete absence of all fungal contaminants from our growth chamber, particularly fungal species occurring in low frequencies.

### Endophyte isolation and culture

We only isolated fungal endophytes from seedlings that had reached their first true leaf stage and were at least 12 cm high eight days after planting. A total of 582 seedlings met these conditions (Table 1). These seedlings were surface-sterilized in bulk following protocols developed by our research team (Greenfield *et al.* 2015). Each seedling was first vigorously washed for 2 min in 0.05 % Triton X-100, then immersed for 2 min in a solution of 0.5 % sodium hypochlorite with 0.05 % Triton X-100, followed by a 1 min immersion in 70 % ethanol, and three separate rinses in sterile distilled water.

**Fig 1:**
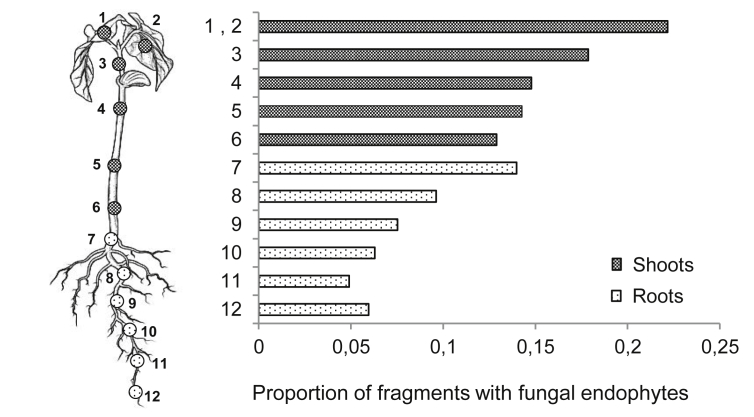
Colonization of fungal endophytes (per part sampled) in common bean seedlings. Sterilized seeds were germinated in sterile vermiculite in a growth chamber and the resulting seedlings sampled for fungal endophytes eight days later. The circles in the seedling illustration point to the location of the 12 fragments taken from each seedling to assess endophyte colonization and localization. The two leaf samples, 1 and 2, were evaluated separately but analytically treated as the same plant part. Shoots includes leaves and stem samples. The figure summarizes data on all 11 cultivars evaluated.

Under sterile conditions in a laminar flow cabinet, we cut each sterilized seedling twice, separating its roots, stem, and leaves. To assess sterilization success, each part was separately imprinted onto a separate PDA media plate thereafter incubated at 26 °C for 10 d (Schulz *et al.* 1998). The three parts were subsequently cut to yield 12 fragments per seedling, as indicated in Fig. 1. Root and stem fragments were 5 mm long and leaf fragments were 5 mm × 5 mm. The disinfection was considered successful if the PDA imprint resulted in no fungal growth by the end of its incubation period. Otherwise, we discarded all fragments corresponding to a contaminated seedling part, maintaining only fragments corresponding to successfully sterilized parts.

After imprinting the fragments, we transferred them onto ¾-strength PDA media plates with penicillin (100 mg L^−1^), streptomycin (200 mg L^−1^), and tetracycline (50 mg L^−1^). The plates were incubated at 26 °C in darkness, and evaluated for fungal growth ensuing from the edges of the fragments for up to 14 d. Such fungal growth was considered ‘endophytic,’ and it was serially sub-cultured onto fresh PDA media with antibiotics (as above) to obtain monosporic cultures (Parsa *et al.* 2013). We cataloged these cultures following the morphospecies approach (Arnold et al., 2000, Crozier et al., 2006, Thomas et al., 2008), based on multiple characters including the colour of the fungal colony, colour changes in the PDA media after fungal growth, the development and organization of the aerial mycelium, the surface texture of the mycelium, the characteristics of the colony margin, and the production of spores. Fungal cultures were deposited in the Fungal Entomopathogen and Endophyte Collection at CIAT.

### DNA extraction, amplification and sequencing

Fungal tissue was obtained by scraping mycelium from the monosporic cultures followed by lyophilization and then maceration with liquid nitrogen in a sterile mortar. One gram of the resulting powdered mycelium was used for DNA extraction using the Invitrogen Easy-DNA extraction kit (Invitrogen Life Technologies, Carlsbad, CA). The nucleic acid concentration of each sample was quantified using a NanoDrop 2000c spectrophotometer (Thermo Scientific, Wilmington, DE) (Desjardins & Conklin 2010), in order to generate a 50 ng ml^−1^ diluted sample. From this dilution, 2 μl was added to 8 μl of a PCR reaction mixture consisting of 0.5 U μl^−1^ Platinum^®^ Taq DNA polymerase (Invitrogen Life Technologies, Carlsbad, CA), 1X PCR Buffer (Invitrogen Life Technologies, Carlsbad, CA), 2 mM Mg^2+^, 0.2 mM dNTP's, and 0.1 pmol μl^−1^ primer (both forward and reverse; see below). The PCR amplification was conducted in a Mastercycler Pro thermal cycler (Eppendorf, Hauppauge, NY) as follows: an initial denaturation step consisting of 2 min at 95 °C; 35 cycles of 30 s at 94 °C, 1 min at 53 °C, 1 min at 72 °C, and a final extension of 5 min at 72 °C.

The PCR products were run on 1.5 % (w/v) agarose gel using 1X boric acid-NaOH buffer stained with SYBR^®^ Safe (Invitrogen Life Technologies, Carlsbad, CA) to visualize the amplification of the desired band length (550–600 bp). The ligation protocol of the PCR products was performed using the Promega ligation protocol (Promega 2015). PCR products were then cloned using the pGEM^®^-T Easy Vector System (Promega, Madison, WI), and transformed into competent cells from *Escherichia coli* colony DH5α (Invitrogen Life Technologies; Carlsbad, CA). The plasmid containing the fragment of interest was purified from *E. coli* and sent to Macrogen Inc. (Gasan-dong, Seoul, Korea) for sequencing. The endophytic fungal isolates were identified by sequencing the internal transcribed ITS region of the rDNA, using universal fungal primers ITS4 (5′TCC TCC GCT TAT TGA TAT GC-3′) for the forward primer and ITS5 (5′GGA AGT AAA AGT CGT AAC AAG G-3′) for the reverse primer (White *et al.* 1990).

The raw sequences received from Macrogen Inc. were edited and assembled using Sequencher Software v5.0 (Gene Codes, MI, USA). For the endophyte identification, the sequences were matched in the GenBank nucleotide database using the Basic Local Alignment Search Tool (BLAST) (Altschul *et al.* 1990). DNA sequences were deposited in GenBank (Table 2).

### Statistical analyses

Fungal endophytes were tabulated and summarized using isolation percentages for each cultivar and plant fragment. For analysis, fragments were grouped into two plant parts: shoots (leaves and stem) and roots. Presence or absence of any fungal endophyte colonization was determined within each plant part summarizing across all fragments. The extent of fungal endophyte colonization was determined within each plant part by the proportion of fragments with colonization. To assess both the distribution of any fungal endophyte colonization and the extent of fungal endophyte colonization across plant parts and cultivars, separate binomial mixed effect models were fit for each with fixed effects for cultivar, plant part, and cultivar by plant part interaction and with a random effect for seed. Post hoc test of simple effects within interaction terms were corrected for multiplicity using the Holm-Bonferroni method. Binomial mixed effect models were fit with the ‘lme4’ package (Bates *et al.* 2013) using R Version 3.1.1 (R Core Team 2014). Post hoc tests were performed using the ‘phia’ package (De Rosario-Martinez 2013).

## Results

A total of 394 fungal endophytes were isolated from 582 seedlings (6924 fragments) belonging to 11 common bean cultivars. Based on their morphological characteristics, these were initially classified into 51 morphospecies. Results from BLAST analyses re-classified them into 42 taxa (Table 2). Only 19 of the taxa were identified to putative species, based on sequence identities of ≥98 % with known GenBank accessions. The remaining taxa were identified to genus or higher levels based on sequence identities of ≥99 % with known GenBank accessions, except for *Fusarium* sp. 5, which shared 91 % sequence identity with its closest GenBank match.

**Fig 2:**
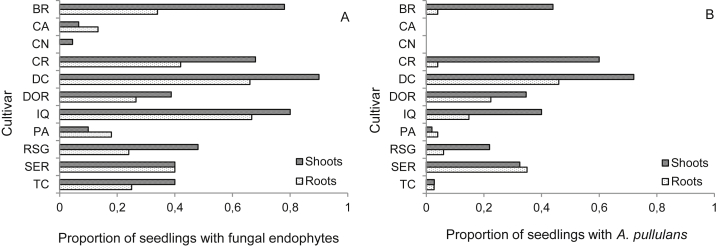
Colonization of fungal endophytes in common bean seedlings from 11 cultivars. Sterilized seeds were germinated in sterile vermiculite in a growth chamber and the resulting seedlings sampled for fungal endophytes eight days later. Shoots includes leaves and stem samples. Cultivar: BR = Bola Roja; CA = Caraota; CN = Cabeza Negra; CR = Cargamanto Rojo; DC = Diacol Calima; DOR = Negro Tacana; IQ = ICA Quimbaya; PA = Palomito; RSG = Radical San Gil; SER = Ser-16; TC = Tio Canela. (**A**). All fungal endophytes. (**B**). Distribution of the most common endophyte, *Aureobasidium pullulans*, among the 11 cultivars.

With the exception of the basidiomycete *Marasmius* aff. *nigrobrunneus*, all other fungal isolates identified were ascomycetes. The most common fungal endophyte was *Aureobasidium pullulans*, found in 46.7 % of all seedlings evaluated (Table 2, Fig 2B). Also common were *Fusarium oxysporum*, *Xylaria* sp., and *Cladosporium cladosporioides*, found in 13.4 %, 11.7 %, and 7.6 % of seedlings, respectively (Table 2). The remaining fungal endophytes were rare, i.e., isolated in less than 2 % of the seedlings evaluated. None of the endophytic fungi reported in our study were recovered during routine inspections for airborne fungal spores in the growth chambers. We therefore infer that fungal endophyte isolates obtained from seedlings likely originated from seeds.

**Fig 3:**
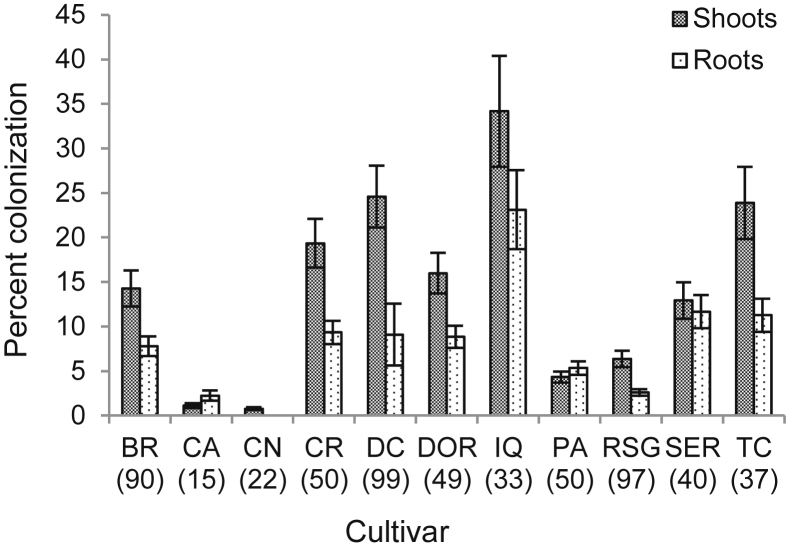
Percent colonization of fungal endophytes in common bean seedlings from 11 cultivars. Seeds were germinated in sterile vermiculite in a growth chamber at 25 °C, and the resulting seedlings sampled for fungal endophytes eight days later. Percent colonization was computed based on the number of 12 fragments per seedling that presented fungal growth. Shoots includes leaves and stem samples. Cultivar: BR = Bola Roja; CA = Caraota; CN = Cabeza Negra; CR = Cargamanto Rojo; DC = Diacol Calima; DOR = Negro Tacana; IQ = ICA Quimbaya; PA = Palomito; RSG = Radical San Gil; SER = Ser-16; TC = Tio Canela. The standard error is represented by the error bars.

Fungal endophytes were more likely to occur in seedling shoots than in roots (Fig 1, Chi^2^ = 29.43, df = 1, *P* = 0.0000) and in some cultivars compared with others (Fig 2A, Chi^2^ = 74.51, df = 10, *P* = 0.0000), with no interaction between seedling part and cultivar (Chi^2^ = 14.23, df = 10, *P* = 0.1628). On average, the extent of colonization was also greater in shoots (Fig 3, Chi^2^ = 98.49, df = 1, *P* < 0.001), and varied among cultivars (Fig 3, Chi^2^ = 76.03, df = 10, *P* < 0.001). In this case, however, the extent of colonization on seedling parts changed depending on the cultivar (Chi^2^ = 25.17, df = 10, *P* = 0.0050). Specifically, cultivars CA, CN, PA, and SER did not show significant differences between shoots and roots while the remaining cultivars showed higher extent of colonization in shoots. On the logistic scale, seed-to-seed variance was estimated at 1.7 for endophyte occurrence and 2.2 for endophyte colonization extent, representing approximately 4 % and 2 % of their respective variations. Hence, the proportion of variation explained by the seeds, including their source or origin, is small relative to other effects.

## Discussion

The objective of this study was to identify fungal endophytes naturally occurring in germinated seeds of the common bean. To our knowledge, this is the first study to document seed-borne fungal diversity in this crop within its center of origin.

The survey detected a low incidence of seed-transmitted common bean pathogens. The only exception was *Fusarium oxysporum*, which occurred in 13.4 % of seedlings evaluated. Other potential pathogens were rare, found in less than 2 % of seedlings evaluated. The most important include *Colletotrichum lindemuthianum*, *Fusarium solani*, *Macrophomina phaseolina*, causing agents of bean anthracnose, *Fusarium* root rot, and ashy stem blight, respectively (Schwartz & Corrales 1989). The relative abundance of *Fusarium* spp. compared to other seed-transmitted plant pathogens was also found in numerous surveys of mycotoxin producing fungi in common bean seeds (Tseng et al., 1995, Castillo et al., 2004, Domijan et al., 2005, Embaby and Abdel-Galil, 2006, El-Samawaty et al., 2014). Although more commonly plant pathogens, some members of the *Fusarium* genus have shown potential as beneficial endophytes against insects and nematodes (Vu et al., 2006, Paparu et al., 2008). Because we evaluated only healthy bean seedlings, the potential exists that some of our *Fusarium* isolates may serve as beneficial endophytes.

More promisingly, close to half of the seedlings we evaluated were endophytically colonized by *Aureobasidium pullulans*. We were unable to find any other report of this species occurring endophytically in common bean seeds. Unlike *Fusarium* members, *A. pullulans* has demonstrated no major pathogenic potential in our target crop or any other cultivated plant. Commonly known as black yeast, *A. pullulans* is an ubiquitous saprophyte in plants (Cooke, 1959, Webb and Mundt, 1978), with demonstrated biological control activity against leaf pathogens (van den Heuvel, 1969, McCormack et al., 1995, Dik and Elad, 1999, Dik et al., 1999; ) and postharvest rots (Bhatt and Vaughan, 1962, Lima et al., 1997, Schena et al., 1999, Ippolito and Nigro, 2000, Schena et al., 2003, Elmer and Reglinski, 2006). Relevantly, a study that applied *A. pullulans* on the surface of bean leaves found that it inhibited leaf lesions caused by *Alternaria zinniae* (van den Heuvel 1969). *Aureobasidium pullulans* has also been reported as a common endophyte in numerous plants (Pugh and Buckley, 1971, Johnson and Whitney, 1989, Schena et al., 2003, Suryanarayanan et al., 2005, Elmer and Reglinski, 2006, Osono, 2008, Martini et al., 2009). Recently, endophytic *A. pullulans* has been implicated in resistance to insect pests (Albrectsen *et al.* 2010) and plant pathogens (Miles *et al.* 2012). Particularly promising is its effect on *Rhizoctonia solani* (Miles *et al.* 2012), a major soil-borne pathogen limiting common bean production (Schwartz & Corrales 1989). Based on its widespread endophytic colonization in our seed samples, and its demonstrated biological control potential, *A. pullulans* could be a promising candidate for the endophytic control of common bean pests and pathogens.

The results also suggest significant differences exist in fungal endophyte compatibility across common bean cultivars. The cultivar Diacol Calima ranked amongst the most compatible, as suggested by its high endophytic colonization levels. This finding is particularly significant to our efforts since Diacol Calima is one of the most important common bean cultivars in Latin America (Voysest 2000), and it is also highly susceptible to several key pathogens, including bean anthracnose, angular leaf spot and root rot (Carlos Jara, pers. comm.). Efforts to evaluate the potential of *A. pullulans* as a disease-inhibiting endophyte in Diacol Calima are therefore justified.

We also found differences in the transmission of seed-borne endophytes across seedling parts. Save a few exceptions, fungal endophytes were more prevalent in shoots than in roots, with the highest colonization occurring in the first true leaves. This distribution may partly reflect the epigeal germination of bean seeds, which renders most of the seed biomass and food reserves above ground. This pattern could also result from plant root and leaf tissues differentially protecting endophytes in the surface sterilization process. A potential implication is that seed-borne endophtyes in the common bean may be more effective for the control of foliar relative to root insect pests and pathogens.

Other papers have reported on endophyte diversity within different plant cultivars, e.g., rice (Fisher & Petrini 1992), wheat (Crous *et al.* 1995), ginseng (Park *et al.* 2012), grapevine (Cosoveanu *et al.* 2014), and cotton (Li *et al.* 2014). All of these articles characterized mature plants grown in an open environment, with prolonged opportunities for fungal invasion after germination. Accordingly, their colonization patterns are unlikely to reflect how seed-borne fungal endophytes are transmitted to seedlings, which is the focus of our contribution.

In summary, the survey of seed-borne fungal endophytes in the common bean revealed *A. pullulans* as the dominant species. When considered together with the published literature, our results suggest endophytic *A. pullulans* could offer significant potential to enhance common bean production as an addition to integrated pest management programs. Future empirical work should focus on seed inoculation trials to experimentally test its endophytic biological control potential in the common bean.

## Figures and Tables

**Table 1:** Sources (and number) of common bean seeds evaluated for fungal endophyte colonization.

Cultivar	CIAT	Markets
Bolon Rojo (BR)	40	50
Caraota (CA)	0	15
Cabeza Negra (CN)	0	22
Cargamanto Rojo (CR)	0	50
Diacol Calima (DC)	50	49
Negro Tacana (DOR)	49	0
ICA Quimbaya (IQ)	33	0
Palomito (PA)	0	50
Radical San Gil (RSG)	47	50
SER-16 (SER)	40	0
Tio Canela (TC)	37	0

**Table 2:** Percentage of common bean seedlings colonized by fungal endophytes. Sterilized seeds were germinated in sterile vermiculite in a growth chamber and the resulting seedlings (n = 582) sampled for fungal endophytes eight days later.

Endophyte ID	GenBank accession number	Cultivar^a^ (number of seedlings)										
BR (90)	CA (15)	CN (22)	CR (50)	DC (99)	DOR (49)	IQ (33)	PA (50)	RSG (97)	SER (40)	TC (37)
*Acremonium* sp.	KR012891	–	–	–	–	2	–	3	–	–	–	–
*Alternaria* sp.	KR012902	1	–	–	–	–	–	–	–	1	–	–
*Aspergillus ustus*	KR012899	–	–	–	–	1	–	–	–	–	–	–
*Aureobasidium pullulans*	KR012884	26	–	–	60	38	37	42	6	13	45	5
*Chaetomium* sp.	KR012907	1	–	–	–	–	–	–	2	–	–	–
*Chaetomium globosum*	KR012922	–	–	–	–	–	–	–	–	2	–	–
*Cladosporium cladosporioides*	KR012880	–	–	5	–	2	–	–	–	–	–	–
*Cladosporium cladosporioides*	KR012883	7	–	–	2	9	2	3	4	3	3	3
*Cladosporium cladosporioides*	KR012897	–	–	–	–	1	–	–	–	–	–	–
*Cochliobolus lunatus*	KR012881	–	–	–	4	1	–	–	–	–	–	–
*Colletotrichum lindemuthianum*	KR012909	2	–	–	–	–	–	–	–	–	3	–
*Curvularia* sp.	KR012919	–	–	–	2	–	–	–	–	–	–	–
*Curvularia affinis*	KR012898	–	–	–	–	1	–	–	–	–	–	–
*Epicoccum* sp.	KR012889	–	–	–	–	1	–	–	–	–	–	–
*Epicoccum nigrum*	KR012895	–	–	–	–	1	–	–	2	–	–	–
*Fusarium* sp. 1^b^	KR012920	2	–	–	–	–	–	–	–	–	–	–
*Fusarium* sp. 2	KR012890	1	–	–	–	–	–	–	–	1	–	–
*Fusarium* sp. 3	KR012894	–	–	–	–	1	–	–	–	–	3	–
*Fusarium* sp. 4	KR012901	1	7	–	–	2	–	–	–	–	–	–
*Fusarium* sp. 5	KR012926	1	–	–	–	–	–	–	–	–	–	–
*Fusarium phaseoli*	KR012896	–	–	–	–	1	–	–	–	–	–	–
*Fusarium oxysporum*	KR012886	4	–	–	–	7	–	48	–	–	–	19
*Fusarium solani*	KR012915	–	7	–	2	–	–	–	–	–	–	–
*Macrophomina phaseolina*	KR012878	–	–	–	–	1	4	3	–	–	–	–
*Marasmius* aff. *nigrobrunneus*	KR012906	1	–	–	–	–	–	–	–	–	–	–
*Neurospora* sp.	KR012910	–	–	–	–	–	–	–	–	1	–	–
*Penicillium commune*	KR012904	1	–	–	–	–	–	–	–	–	–	–
*Pestalotiopsis* sp.	KR012882	1	–	–	–	–	–	–	–	–	–	–
*Pestalotiopsis microspora*	KR012928	1	–	–	–	2	–	–	–	1	–	–
*Pestalotiopsis sydowiana*	KR012887	–	–	–	–	–	–	3	–	–	3	–
*Pestalotiopsis* sp.	KR012893	–	–	–	–	1	–	–	–	–	3	–
*Peyronellaea glomerata*	KR012905	1	–	–	–	–	–	–	4	–	–	–
*Phaeosphaeriopsis* sp.	KR012892	–	–	–	2	–	–	–	–	1	3	–
*Pleospora* sp.	KR012918	1	–	–	–	–	–	–	–	1	–	–
*Stemphylium* sp.	KR012908	–	–	–	–	1	–	–	–	1	–	–
*Stemphylium solani*	KR012916	1	–	–	–	–	–	–	–	–	–	–
*Talaromyces* aff. *verruculosus*	KR012927	–	–	–	2	1	–	–	–	–	–	–
Uncultured ascomycete	KR012903	1	–	–	2	4	–	–	–	–	–	–
Uncultured *Aureobasidium*	KR012885	8	7	–	34	14	2	18	12	13	3	3
Uncultured endophytic fungus	KR012923	–	–	–	–	–	–	–	–	1	–	–
Uncultured *Xylariales*	KR012888	2	–	–	–	2	–	–	2	–	–	3
*Xylaria* sp.	KR012879	6	–	–	–	–	–	36	–	2	–	24

a BR = Bola Roja; CA = Caraota; CN = Cabeza Negra; CR = Cargamanto Rojo; DC = Diacol Calima; DOR = Negro Tacana; IQ = Ica. Quimbaya; PA = Palomito; RSG = Radical San Gil; SER = Ser-16; TC = Tio Canela.

b Numbers in *Fusarium* endophytes ID correspond to isolates that presented different sequences length and different identity percent in BLAST analysis.
